# Supplementary material for: Three Varieties of Grape Pomace, with Distinctive Extractable:Non-Extractable Polyphenol Ratios, Differentially Reduce Obesity and Its Complications in Rats Fed a High-Fat High-Fructose Diet
Source: Foods. 2023 Mar 23;12(7):1370. doi: 10.3390/foods12071370 (PMC10093191; doi:10.3390/foods12071370)
Supplement: Supplementary file 1 [file foods-12-01370-s001.zip › foods-2258326-supplementary.pdf]

Table S1. Retention time (min), formula, fragments, and adducts of polyphenols identified.

Component name	RT (min)	Formula	Expected mass (Da)	Observed m/z	Mass error (ppm)	Fragments	Adducts
(Iso)-rhamnetin*	9.08	$C_{16}H_{12}O_7$	316.0583	317.0646	-2.9344	302.04160, 153.01760	$[M+H]^+$
Peonidin dihexoside	3.83	$C_{28}H_{33}O_{16}$	625.1769	625.1791	4.4748	301.07079	$[M]^+$
Syringetin hexoside	6.53	$C_{23}H_{24}O_{13}$	508.1217	509.1277	-2.4476	347.07648, 153.01758	$[M+H]^+$
Malvidin dihexoside	3.89	$C_{29}H_{35}O_{17}$	655.1874	655.1849	-3.0535	331.08113	$[M]^+$
Delphinidin rutinoside	6.02	$C_{27}H_{31}O_{16}$	611.1401	611.1404	1.4082	303.04963	$[M]^+$
Malvidin caffeoyl-hexoside	6.25	$C_{32}H_{31}O_{15}$	655.1663	655.1667	1.5089	331.08110	$[M]^+$
Malvidin hexoside pyruvic acid (Vitisin A)	5.23	$C_{26}H_{25}O_{14}$	561.1244	561.1253	2.4976	331.08087	$[M]^+$
Malvidin coumaroyl-hexoside	6.76	$C_{32}H_{31}O_{14}$	639.1714	639.1713	0.8022	331.08097	$[M]^+$
(Epi)-catechin hexoside isomer II	3.86	$C_{21}H_{24}O_{11}$	452.1319	451.1277	6.8173	289.1239	$[M-H]^-$
Delphinidin acetyl-hexoside	5.03	$C_{23}H_{23}O_{13}$	507.1139	507.1138	0.8701	303.04964	$[M]^+$
Malvidin hexoside	4.74	$C_{23}H_{25}O_{12}$	493.1346	493.1339	-0.3280	331.08059	$[M]^+$
Petunidin acetyl-hexoside	5.46	$C_{24}H_{25}O_{13}$	521.1295	521.1292	0.4246	317.06522	$[M]^+$
(Iso)-rhamnetin rhamnoside	4.87	$C_{22}H_{22}O_{12}$	478.1111	479.1199	3.0470	317.06491, 302.04184, 153.01768	$[M+H]^+$
Quercetin hexoside	3.70	$C_{21}H_{20}O_{12}$	464.0955	465.1012	-3.2789	303.04906, 153.01782	$[M+H]^+$
Delphinidin hexoside	3.70	$C_{21}H_{21}O_{12}$	465.1033	465.1025	-0.5392	303.04906	$[M]^+$
Malvidin acetyl-hexoside	5.86	$C_{25}H_{27}O_{13}$	535.1452	535.1448	0.2899	331.08097	$[M]^+$
(Iso)-rhamnetin hexoside	4.30	$C_{22}H_{22}O_{12}$	478.1111	479.1168	-3.4171	317.06477, 302.04152	$[M+H]^+$
Petunidin hexoside	4.29	$C_{22}H_{23}O_{12}$	479.1190	479.1182	-0.4743	317.06477	$[M]^+$
Quercetin*	7.87	$C_{15}H_{10}O_7$	302.0427	303.0487	-3.9394	153.0173	$[M+H]^+$
Kaempferol*	8.87	$C_{15}H_{10}O_6$	286.0477	287.0542	-2.7255	153.0171	$[M+H]^+$
Cyanidin acetyl-hexoside	5.39	$C_{23}H_{23}O_{12}$	491.1190	491.1195	2.3154	287.05473	$[M]^+$
(+)-Catechin*	3.75	$C_{15}H_{14}O_6$	290.0790	289.0712	-2.0841	245.08094, 109.02932	$[M-H]^-$
(-)-Epicatechin*	4.64	$C_{15}H_{14}O_6$	290.0790	289.0726	2.9018	245.08093, 125.02390	$[M-H]^-$
(Epi)-catechin gallate	5.78	$C_{22}H_{18}O_{10}$	442.0900	441.0835	1.7720	289.07112, 169.01390, 125.02404	$[M-H]^-$
Cinnamtannin A3	5.14	$C_{75}H_{62}O_{30}$	1442.3326	1441.3354	6.9767	1154.27308, 577.13570, 287.07071	$[M-H]^-$

Procyanidin trimer C (cinnamtannin A1) isomer I	4.00	$C_{45}H_{38}O_{18}$	866.2058	865.2031	5.2814	577.12242, 287.05602	$[M-H]^-$
Procyanidin trimer C (cinnamtannin A1) isomer II	4.42	$C_{45}H_{38}O_{18}$	866.2058	865.2036	5.8299	577.12159, 287.05582	$[M-H]^-$
Prodelphinidin tetramer (cinnamtannin A2)	5.05	$C_{60}H_{50}O_{24}$	1154.2692	1153.2717	8.4566	289.07071, 577.13570, 864.69765	$[M-H]^-$
Kaempferol hexoside	4.13	$C_{21}H_{20}O_{11}$	448.1006	449.1079	0.2379	287.0545	$[M+H]^+$
Cyanidin hexoside	4.12	$C_{21}H_{21}O_{11}$	449.1084	449.1077	-0.3352	287.05454	$[M]^+$
Peonidin hexoside	4.67	$C_{22}H_{23}O_{11}$	463.1240	463.1236	0.2961	301.07024	$[M]^+$
Gallic acid hexoside	1.42	$C_{13}H_{16}O_{10}$	332.0743	331.0678	2.3543	289.07308, 245.08518	$[M-H]^-$
Procyanidin dimer B (EC-EC) isomer I	3.43	$C_{30}H_{26}O_{12}$	578.1424	577.1393	7.1895	289.0708	$[M-H]^-$
Malvidin arabinoside	5.24	$C_{22}H_{23}O_{11}$	463.1240	463.1243	1.7447	331.08087	$[M]^+$
Procyanidin dimer B (EC-EC) isomer II	4.33	$C_{30}H_{26}O_{12}$	578.1424	577.1376	4.2239	289.0704	$[M-H]^-$
Procyanidin trimer C (cinnamtannin A1) isomer III	4.92	$C_{45}H_{38}O_{18}$	866.2058	865.2043	6.6591	577.13595, 287.05537	$[M-H]^-$
